# Association between serum progesterone levels on the day of embryo transfer and clinical pregnancy outcomes in POSEIDON Group 1 patients

**DOI:** 10.3389/fendo.2026.1766243

**Published:** 2026-05-28

**Authors:** Xin Wang, Lili Zhuang, Luqing Zhang, Zhenteng Liu, Huishan Zhao, Dongmei Zhao, Yingqian Peng, Hongchu Bao

**Affiliations:** Department of Reproductive Medicine, Qingdao University Medical College Affiliated Yantai Yuhuangding Hospital, Yantai, China

**Keywords:** clinical pregnancy rate, dydrogesterone, embryo transfer, serum progesterone, vaginal progesterone

## Abstract

**Objective:**

To investigate the relationship between serum progesterone (P) on the day of embryo transfer (ET) and pregnancy outcomes in patients classified as POSEIDON Group 1.

**Methods:**

This retrospective cohort study was conducted at the Reproductive Center of Yantai Yuhuangding Hospital between January 2016 and December 2023, enrolling 402 patients undergoing single or double ET on day 3 or day 5. Serum P and estradiol (E_2_) were measured on the day of ET, with clinical pregnancy rate as the primary outcome. Patients were stratified into three groups based on the 10th and 90th percentiles of serum P. Categorical variables were compared using the Pearson chi-square test or Fisher’s exact test, continuous variables using Student’s t-test, and multiple quantitative data using one-way analysis of variance. Multivariate logistic regression was performed to adjust for potential confounders. Additionally, sensitivity analysis and a robustness test based on body mass index (BMI) stratification were conducted.

**Results:**

The 10th and 90th percentiles of serum P on the day of ET were 9.45 ng/mL and 23.32 ng/mL, respectively. The clinical pregnancy rate was highest in the 9.45-23.32 ng/mL group and decreased significantly when serum P exceeded 23.32 ng/mL. After adjusting for confounders, multivariate logistic regression demonstrated that high serum P (≥23.32 ng/mL) was independently associated with a lower clinical pregnancy rate (adjusted OR = 1.454, 95% CI: 1.032-2.048, *P* = 0.032). Sensitivity analysis confirmed the robustness of this association. BMI stratification revealed that elevated serum P was associated with a reduced clinical pregnancy rate in normal-weight patients, but not in overweight/obese patients, with a significant interaction between serum P and BMI.

**Conclusions:**

Serum P on the day of ET was significantly associated with clinical pregnancy rate in POSEIDON Group 1 patients, and BMI significantly modified this association. High serum P was linked to a lower clinical pregnancy rate in normal-weight patients, but not in overweight/obese patients. These findings support the implementation of individualized luteal support strategies based on serum P and BMI stratification.

## 1 Introduction

In recent years, with advances in embryo cryopreservation technology and the growing number of patients undergoing preimplantation genetic testing (PGT) and fertility preservation, the number of frozen embryo transfer (FET) cycles has increased worldwide. This trend has simultaneously reduced the risk of late-onset ovarian hyperstimulation syndrome (OHSS) associated with fresh cycles and the adverse effects of supraphysiological serum estradiol (E_2_) on endometrial receptivity (1). Progesterone (P) is indispensable for endometrial transformation, embryo implantation, and pregnancy maintenance. Compared with natural and stimulated cycles, hormone replacement therapy (HRT) cycles for artificial endometrial preparation have gained wider acceptance, as they permit control over the duration of exogenous P exposure and allow precise scheduling of the transfer day to achieve optimal embryo-endometrial synchronization (2). In artificial cycles, endogenous P secretion is absent; thus, adequate exogenous P supplementation is crucial for successful embryo transfer (ET). HRT cycles mimic physiological menstrual cycles: estrogen is administered from day 2 to 3 of menstruation until endometrial thickness reaches 8 mm, followed by P conversion for endometrial preparation.

It has been reported that in natural cycles, elevated serum P before ovulation triggers premature luteinization and compromised endometrial receptivity, ultimately leading to reduced embryo implantation rates (3). Conversely, insufficient P supplementation prior to ET also exerts detrimental effects on embryo implantation rates (4). P can be administered via multiple routes, including vaginal, intramuscular, subcutaneous, rectal, and oral routes, all of which present distinct pharmacokinetic profiles. Several researchers have proposed that intrauterine P concentrations are more crucial for successful pregnancy outcomes compared with serum P (5). However, other studies have demonstrated that the systemic anti-inflammatory effects of serum P are equally important (6), and endometrial P is mainly derived from arterial blood (7). Currently, no consensus has been reached regarding the optimal route, dosage, and duration of P administration in FET cycles, all of which directly influence serum P on the day of ET. Previous studies have suggested that higher serum P on the day of ET is associated with better pregnancy outcomes (8). Nevertheless, other studies have identified an optimal threshold range for serum P on the day of ET, suggesting that both excessively low and excessively high serum P exert detrimental effects on pregnancy outcomes (9).

Given this background, we aimed to evaluate the association between serum P on the day of ET and pregnancy outcomes. The present study specifically focused on POSEIDON Group 1 (women aged ≤35 years with normal ovarian reserve and unexpected poor ovarian response) patients undergoing HRT cycles with luteal support provided by vaginal micronized P combined with oral dydrogesterone, in order to provide evidence for individualized luteal support strategies in this population.

## 2 Methods

### 2.1 Study design

A retrospective cohort study was conducted at the Reproductive Medicine Center of Yantai Yuhuangding Hospital. Participants were patients undergoing FET in HRT cycles between January 2016 and December 2023.

### 2.2 Study population

A total of 402 infertile patients undergoing their first oocyte retrieval cycle were enrolled in this study. All patients underwent single or double ET at either the cleavage stage or blastocyst stage following artificial endometrial preparation with HRT.

### 2.3 Inclusion and exclusion criteria

#### 2.3.1 Inclusion criteria

Patients were classified as POSEIDON Group 1 according to the POSEIDON criteria, defined as women aged under 35 years with normal ovarian reserve (antral follicle count ≥5 and anti-Müllerian hormone (AMH) ≥1.2 ng/mL) and a previous history of unexpected suboptimal or low ovarian response to standard gonadotropin stimulation. Additional inclusion criteria included a triple-layer endometrium ≥ 8 mm after HRT, and transfer of 1–2 day 5 or day 6 embryos following P initiation.

#### 2.3.2 Exclusion criteria

Patients were excluded if they had a history of recurrent spontaneous abortion, repeated implantation failure (RIF), severe male factor infertility, uterine abnormalities (e.g., septate uterus, unicornuate uterus, adenomyosis, or multiple uterine fibroids), chromosomal abnormalities, immune disorders, or other systemic diseases. RIF was defined as the failure to achieve a clinical pregnancy in women under 40 years of age after transfer of at least three high-quality embryos over three fresh or frozen cycles, including day 3 embryos (≥8 cells, uniform blastomere size, fragmentation rate < 10%) and blastocysts (≥3BB).

### 2.4 Endometrial preparation

All patients underwent endometrial preparation using a step−up estrogen protocol in HRT cycles. On day 2 or 3 of menstruation, patients began oral administration of estradiol valerate (Progynova^®^, Bayer Hispania, Barcelona, Spain) at a daily dose of 2 mg. After 4 days, the dosage was increased to 4 mg daily, and following an additional 4 days, the dose was adjusted to 6 mg daily. After 16 days of estrogen administration, a transvaginal 2D ultrasound was performed to assess endometrial thickness and confirm a triple−layer pattern. Serum E_2_ and P were measured simultaneously to rule out spontaneous ovulation. ET was scheduled when endometrial thickness was ≥ 8 mm, a triple−layer pattern was present, and serum P was < 1.0 ng/mL. Luteal phase support (LPS) was initiated with vaginal micronized P (Utrogestan^®^, Cyndea Pharma, S.L., Spain) at 200 mg three times daily and oral dydrogesterone (Duphaston^®^, Abbott Biologicals B.V., Netherlands) at 10 mg twice daily.

### 2.5 Embryo transfer

Day 3 cleavage-stage embryos were transferred on day 5 of P initiation, whereas day 5 or day 6 blastocyst-stage embryos were transferred on day 6 of P initiation. ET was performed using a flexible catheter under transabdominal ultrasound guidance. Following ET, daily estrogen and P supplementation was continued. If serum β−hCG testing was negative on day 14 after ET, hormonal support was discontinued. If a clinical pregnancy was achieved, hormonal treatment was maintained until 10 weeks of gestation.

All ET procedures were performed by the same experienced physician under ultrasound guidance, with consistent use of the same type of transfer catheter and standardized surgical techniques throughout the study. All transfer procedures were uneventful, and all patients exhibited excellent medication compliance with standardized luteal phase support. Embryo quality was assessed in accordance with a standardized grading system, and embryo quality parameters were included as covariates in the subsequent multivariate logistic regression analysis. All embryo culture conditions, vitrification protocols, laboratory assays, and clinical practices remained consistent and unchanged during the entire study period from 2016 to 2023. Accordingly, no temporal confounding associated with protocol alterations existed in this study.

### 2.6 Embryo grading criteria

Cleavage−stage embryos (day 3) were graded according to cell number, blastomere uniformity, and fragmentation rate. High−quality day 3 embryos were defined as those with ≥8 cells, evenly sized blastomeres, and a fragmentation rate <10%. Blastocyst−stage embryos (day 5/6) were graded using the Gardner grading system. High−quality blastocysts were defined as ≥3BB, including adequate inner cell mass and trophectoderm development.

### 2.7 Endpoints

The primary endpoint was the clinical pregnancy rate. Secondary endpoints included the live birth rate, biochemical pregnancy rate, and miscarriage rate. Pregnancy was defined as a positive urinary β-hCG test (β-hCG, Hangzhou AllTest Biotech Co., Ltd., Hangzhou, China) performed 14 days after FET. Clinical pregnancy was defined as visualization of a gestational sac on transvaginal ultrasound at 7–8 weeks of gestation. Biochemical pregnancy was defined as a positive urinary hCG test without subsequent evidence of clinical pregnancy. Miscarriage was defined as pregnancy loss occurring up to 21 + 6 weeks of gestation following a positive pregnancy test. Live birth was defined as the delivery of a live neonate at or after 22 + 0 weeks of gestation.

### 2.8 Progesterone and estradiol measurement

All patients took medications at 6:00 AM, and blood samples were collected at 7:00 AM on the day of ET. Serum E_2_ and P on the day of ET were measured via electrochemiluminescence immunoassay (ECLIA) using a Cobas^®^ e601 analyzer (Roche Diagnostics GmbH, Germany) at the clinical laboratory of our hospital. Serum.

P was expressed in ng/mL, while serum E_2_ was expressed in pg/mL.For serum P detection, the intra-assay coefficient of variation (CV) was 2.3-11.9% and the inter-assay CV was 3.2-22.5% at concentrations ranging from 0.02 to 1.8 ng/mL, with an analytical sensitivity of 0.05 ng/mL. For serum E_2_ detection, the intra-assay CV was 1.1-6.7% and the inter-assay CV was 1.9-10.6%, with a measurement range of 5.0–3000 pg/mL and an analytical sensitivity of 5 pg/mL.

### 2.9 Statistical analysis

Serum P on the day of ET was categorized according to the 5th, 10th, 25th, 50th, 75th, 90th, and 95th percentiles. Patients were further divided into three groups based on serum P, with the 10th and 90th percentiles used as cutoff values. Categorical variables were compared among groups using the Pearson chi−square test or Fisher’s exact test. Student’s t−test was used for comparisons of continuous variables between two groups, and one−way analysis of variance (ANOVA) was applied for comparisons among multiple groups. Clinical pregnancy rate, biochemical pregnancy rate, live birth rate, and spontaneous abortion rate were compared among the three groups. We used the 10th (P10) and 90th (P90) percentiles of serum P distribution as cutoff values to stratify patients into three groups. As univariate analysis showed no significant difference in serum P between pregnant and non-pregnant patients, a valid and clinically meaningful cutoff could not be determined by receiver operating characteristic (ROC) curve analysis. Furthermore, serum P on the day of ET mainly reflects interindividual variability in medication absorption, rather than acting as a diagnostic index with definitive sensitivity and specificity. For these reasons, we adopted percentile-based stratification for subsequent analyses.

Multivariate logistic regression analysis was performed to investigate the independent effect of serum P on clinical pregnancy rate and to identify independent factors associated with clinical pregnancy. The regression model was adjusted for comprehensive potential confounders, including age, body mass index (BMI), AMH, type and duration of infertility, number of oocytes retrieved in the fresh cycle, insemination method in the fresh cycle, endometrial thickness, number of embryos transferred (single versus double), embryo stage (cleavage versus blastocyst), number of high−quality embryos transferred, and serum E_2_. In the binary logistic regression model, continuous variables were directly entered into the model, whereas categorical variables were transformed into dummy variables with a predefined reference group before analysis. Sensitivity analysis was conducted by combining the low and high P groups and comparing them with the middle group. In addition, robustness checks and interaction analyses were performed using BMI as a stratification or interaction variable to verify the stability of the main results.

## 3 Results

### 3.1 Descriptive analysis

Continuous variables are presented as mean ± standard deviation (SD), and categorical variables are presented as number (percentage). Among the 402 patients included, the mean age was 30.70 ± 2.42 years, the mean BMI was 24.34 ± 3.91 kg/m², and the mean AMH was 5.41 ± 4.58 ng/mL. Primary infertility accounted for 64.18% of the cohort, and the mean duration of infertility was 3.65 ± 2.05 years.

### 3.2 Distribution of serum progesterone

**Figure 1:**
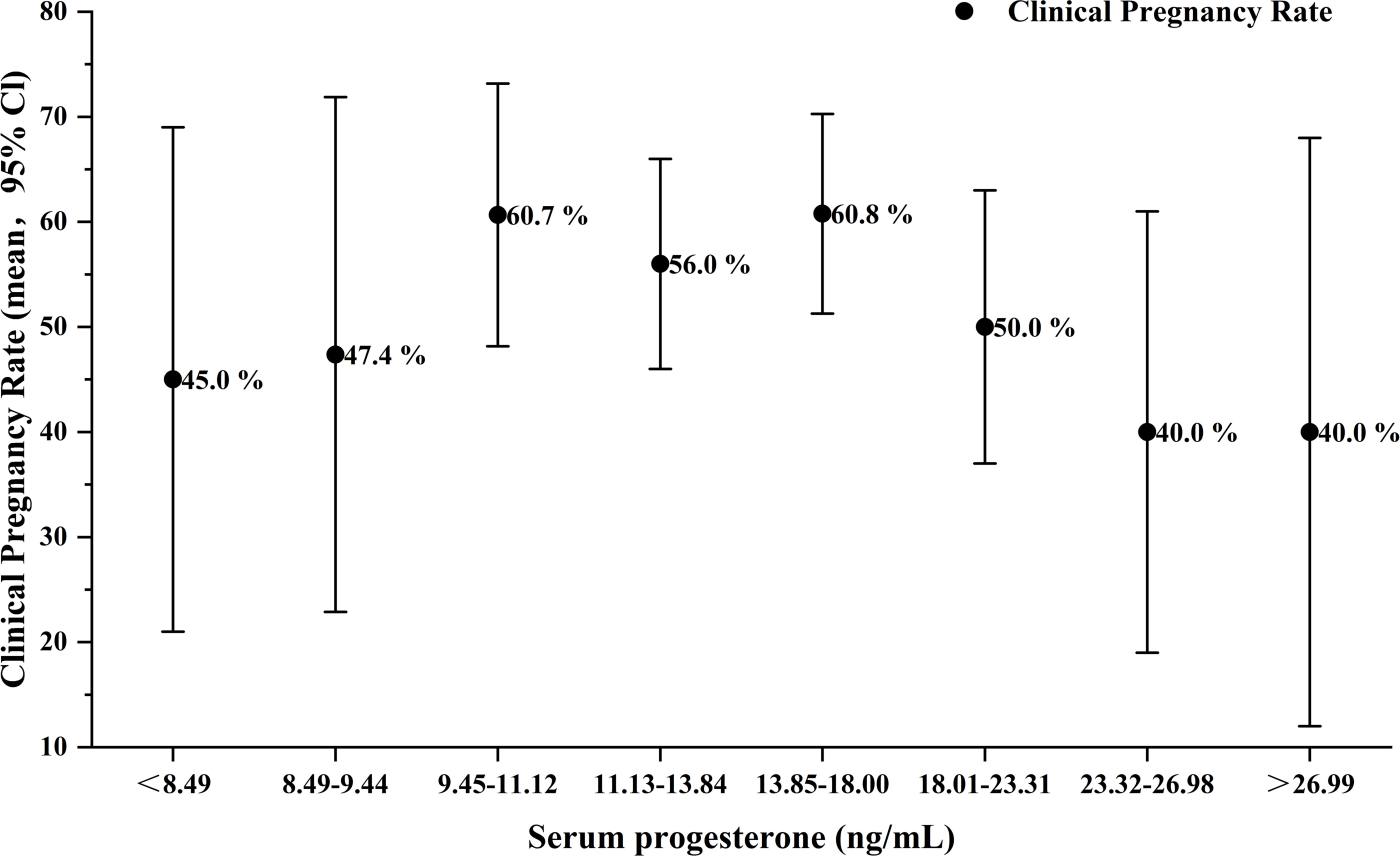
Clinical pregnancy rate by serum progesterone percentile on embryo transfer day. Data: mean, 95% CI.

The percentile values of serum P on the day of ET are presented in **Table 1**. The 5th, 10th, 25th, 50th, 75th, 90th, and 95th percentiles were 8.49, 9.45, 11.13, 13.85, 18.01, 23.32, and 26.99 ng/mL, respectively. As shown in **Figure 1**, the clinical pregnancy rate initially increased with rising serum P, peaking at 60.78% in the 13.85-18.00 ng/mL group, and then declined, reaching 40% in groups with serum P >23.32 ng/mL.

**Table 1:** Percentile distribution of serum progesterone.

Percentiles	P5	P10	P25	P50	P75	P90	P95
Progesterone	8.49	9.45	11.13	13.85	18.01	23.32	26.99

Serum progesterone (ng/mL).

### 3.3 Baseline characteristics of patients

Patients were stratified into three groups based on the 10th (9.45 ng/mL) and 90th (23.32 ng/mL) percentiles of serum P on the day of ET: Group A (P < 9.45 ng/mL, n=39), Group B (9.45≤P < 23.32 ng/mL, n=323), and Group C (P≥23.32 ng/mL, n=40). Baseline characteristics are summarized in **Table 2**. Significant differences were observed in BMI (*P*-value = 0.011) and serum E_2_ on the day of ET (*P*-value = 0.008) among the three groups. No significant differences were found in age, AMH, infertility type/duration, number of oocytes retrieved, insemination method, endometrial thickness, number/type of transferred embryos, or number of high-quality embryos (all *P*-value > 0.05).

**Table 2:** Baseline clinical characteristics by serum progesterone level on embryo transfer day.

Variable	Group A (n=39)	Group B (n=323)	Group C (n=40)	P-value
Age, years	30.77±2.36	30.72±2.41	30.4±2.6	0.712
BMI, kg/m^2^	24.86±3.68	24.48±3.84	22.62±4.3	0.011
AMH, ng/ml	6.44±5.23	5.33±4.44	4.98±4.97	0.298
Infertility type Primary infertility (*n*, %) Secondary infertility (*n*, %)	22 (56.4%) 17 (43.6%)	208 (64.4%) 115 (35.6%)	28 (70%) 12 (30%)	0.173
Infertility years, years	3.36±2.30	3.62±1.96	4.24±2.46	0.125
NO.of eggs in fresh cycle (*n*)	6.26±2.14	6.84±1.85	6.63±1.88	0.173
Fresh cycle insemination IVF (*n*, %) ICSI (*n*, %)	35 (89.7%) 4 (10.3%)	287 (88.9%) 36 (11.1%)	35 (87.5%) 5 (12.5%)	0.956
Intimal thickness, mm	10.61±2.23	10.04±1.71	10.18±1.69	0.151
No.of ET (*n*)	1.33±0.48	1.32±0.47	1.45±0.50	0.236
Embryo Stage Cleavage (*n*, %) blastocyst (*n*, %)	12 (30.8%) 27 (69.2%)	125 (38.7%) 198 (61.3%)	19 (47.5%) 21 (52.5%)	0.311
No.of high-quality embryo (*n*)	0.79±0.70	0.89±0.71	0.90±0.78	0.704
E2 level on ET day, pg/mL	254.33±133.75	272.40±224.69	390.15±347.71	0.008

Continuous variables were expressed as mean±SD and compared using one-way ANOVA. Categorical variables were expressed as n (%) and compared using the Chi-square test. A P-value < 0.05 was considered statistically significant.

### 3.4 Clinical outcomes among progesterone groups

To avoid potential bias caused by arbitrary cutoff values, we analyzed serum P as a continuous variable. After adjustment for confounding factors, results revealed a significant non-linear relationship between serum P and clinical pregnancy rate (*P* for non-linearity < 0.05), confirming the adverse effect of elevated serum P on clinical pregnancy outcomes.

The clinical pregnancy rates among the three subgroups were compared and are shown in **Table 3**. Pairwise comparisons revealed a significant difference between Group B and Group C (*P* = 0.046). Detailed clinical outcomes, including live birth rate, biochemical pregnancy rate, and spontaneous abortion rate, are presented in **Table 4**. Clinical pregnancy rates were 46.15% in Group A, 56.70% in Group B, and 40.00% in Group C, with a borderline significant difference among the three groups (*P* = 0.08). Live birth rates were 38.46%, 46.44%, and 35.00% in Groups A, B, and C, respectively (*P* = 0.282). Biochemical pregnancy rates were 15.38%, 7.12%, and 2.50% in Groups A, B, and C, respectively (*P* = 0.081). Spontaneous abortion rates were 7.69%, 9.91%, and 5.00% in Groups A, B, and C, respectively (*P* = 0.564).

**Table 3:** Comparison of clinical pregnancy rates among three progesterone subgroups on the day of embryo transfer.

Outcome	Group A (n=39)	Group B (n=323)	Group C (n=04)	P-value
Clinical pregnancy rate	18 (46.15%)	183 (56.70%)	16/40 (40.00%)	0.08
P	0.212		0.046	

Pairwise comparisons: Group A vs. Group B, P = 0.212; Group B vs. Group C, P = 0.046. Overall comparison among three groups, P = 0.08.

**Table 4:** Comparison of clinical outcomes among three progesterone subgroups on the day of embryo transfer.

Clinical outcomes	Group A (n=39)	Group B (n=323)	Group C (n=40)	P-value
Clinical pregnancy rate (*n*,%)	18 (46.15%)	183 (56.7%)	16 (40%)	0.08
Live birth rate (*n*,%)	15 (38.5%)	150 (46.4%)	14 (35%)	0.282
Biochemical pregnancy rate (*n*,%)	6 (15.38%)	23 (7.12%)	1 (2.5%)	0.081
Spontaneous abortion rate (*n*,%)	3 (7.69%)	32 (9.91%)	2 (5%)	0.564

Clinical outcome rates were compared among three groups using the Chi-square test. A P-value < 0.05 was considered statistically significant.

### 3.5 Multivariate logistic regression analysis

Multivariate logistic regression analysis was performed to identify independent factors associated with clinical pregnancy (**Table 5**). After adjusting for confounders, including age, BMI, AMH, infertility parameters, and embryo characteristics, serum P on the day of ET was an independent predictor of clinical pregnancy (adjusted OR = 1.454, 95% CI: 1.032-2.048, *P* = 0.032). In subgroup analysis using the intermediate serum P group (Group B) as the reference, the low serum P group (Group A) showed a non-significant trend toward reduced clinical pregnancy rates (adjusted OR = 0.596, 95% CI: 0.286-1.240, *P* = 0.166), whereas the high serum P group (Group C) exhibited a significantly lower clinical pregnancy rate (adjusted OR = 0.397, 95% CI: 0.188-0.837, *P* = 0.015), indicating an adverse effect of elevated serum P on pregnancy outcomes. Additionally, the number of high-quality embryos transferred was the strongest positive predictor of clinical pregnancy (adjusted OR = 2.785, 95% CI: 1.929-4.021, *P* < 0.001). Fresh cycle insemination method (ICSI vs. IVF) was also associated with higher clinical pregnancy rates (adjusted OR = 2.031, 95% CI: 1.015-4.065, *P* = 0.045).

**Table 5:** Multivariate logistic regression analysis for clinical pregnancy rate.

Variable	B	Adjusted OR (95% CI)	P-value
Age	-0.086	0.918 (0.834-1.010)	0.080
BMI	-0.026	0.974 (0.921-1.031)	0.365
AMH	0.031	1.032 (0.981-1.085)	0.222
Infertility type	0.237	1.267 (0.784-2.049)	0.334
Infertility years	0.009	1.009 (0.898-1.133)	0.886
NO.of eggs in fresh cycle	0.372	0.927 (0.817-1.051)	0.234
Fresh cycle insemination	0.709	2.031 (1.015-4.065)	0.045
Endometrial thickness	0.692	1.997 (0.589-6.774)	0.267
NO.of transferred embryos	0.372	1.450 (0.555-3.791)	0.448
Embryo Stage	0.854	2.349 (0.938-5.882)	0.068
No.of high-quality embryo	1.024	2.785 (1.929-4.021)	0.000
E2 level on ET day	0.000	1.000 (0.999-1.001)	0.550
P level on ET day	0.375	1.454 (1.032-2.048)	0.032
P level on ET day (Reference: Group B)			
Group A	-0.518	0.596 (0.286–1.240)	0.166
Group C	-0.925	0.397 (0.188–0.837)	0.015

### 3.6 Sensitivity analysis

To evaluate the potential influence of imbalanced sample sizes among the three groups, a sensitivity analysis was performed by combining the low-serum P and high-serum P groups into a single category (**Table 6**). Unadjusted analysis showed no significant difference in clinical pregnancy rate between the combined group and the middle serum P group (χ²= 3.323, *P* = 0.078). However, after adjustment for confounding factors via multivariate logistic regression, serum P remained independently associated with clinical pregnancy rate (OR = 1.745, 95% CI: 1.026-3.066, *P* = 0.040). These results indicated that the association between serum P on the day of ET and clinical pregnancy rate was robust and not significantly affected by the imbalanced sample size.

**Table 6:** Sensitivity analysis of progesterone level and clinical pregnancy rate (for Imbalanced sample size).

Analysis type	Comparison groups	Statistical index	Value	P-value
Unadjusted analysis	Combined group (A + C) vs. B	χ² value	3.323	0.078
Multivariate logistic regression analysisa	Combined group (A + C) vs. B	Adjusted OR (95%CI)	1.745 (1.026–3.066)	0.040

^a^ Adjusted for age, BMI, AMH, infertility type, duration of infertility, number of oocytes retrieved in fresh cycle, fresh cycle insemination, endometrial thickness, number of transferred embryos, embryo stage, number of high-quality embryos, and estradiol level on ET day.

### 3.7 Robustness and interaction analysis (BMI stratification)

To explore potential effect modification by BMI, we performed subgroup analyses stratified by normal weight (BMI < 24 kg/m²) and overweight/obese (BMI ≥ 24 kg/m²) status, in addition to interaction tests (**Table 7**). Among normal-weight patients, the clinical pregnancy rates were 46.15% in Group A, 54.92% in Group B, and 31.03% in Group C, with a significant difference among the three groups (*P* = 0.022). After adjustment for confounders, middle-range serum P was associated with lower odds of clinical pregnancy compared with the combined low and high groups (adjusted OR = 0.400, 95% CI: 0.186-0.859, *P* = 0.019). Among overweight/obese patients, the clinical pregnancy rates were 46.15% in Group A, 56.47% in Group B, and 72.73% in Group C, with no significant difference among the three groups (*P* = 0.341). The adjusted OR for middle-range serum P was 1.665 (95% CI: 0.811-3.421, *P* = 0.165). The interaction term between serum P and BMI was statistically significant (OR = 1.135, 95% CI: 1.003-1.284, *P* = 0.045), indicating that the association between serum P on the day of ET and clinical pregnancy rate differed significantly between normal-weight and overweight/obese patients.

**Table 7:** Comparison of clinical pregnancy rates among normal weight, overweight and obese patients.

BMI subgroup	Group A	Group B	Group C	P-value^a^	Adjusted OR (95% CI), P-value^b^	Interaction OR (95% CI), P-value^c^
Normal Weight	6/13 (46.15%)	84/153 (54.92%)	9/29 (31.03%)	0.022	0.400 (0.186-0.859), 0.019	1.135 (1.003-1.284), 0.045
Overweight and Obese	12/26 (46.15%)	96/170 (56.47%)	8/11 (72.73%)	0.341	1.665 (0.811-3.421), 0.165	

^a^P-value for the comparison of clinical pregnancy rates among progesterone Group A, B and C within each BMI subgroup (one-way ANOVA for categorical outcomes).

^b^Adjusted for age, AMH, infertility type, infertility duration, number of oocytes retrieved in fresh cycle, fresh cycle insemination, endometrial thickness, number of transferred embryos, embryo stage, number of high-quality embryos, and serum estradiol level on the day of ET.

^c^Interaction OR for the association between serum progesterone levels, BMI and clinical pregnancy rate (adjusted for the above confounding factors).

## 4 Discussion

In the present study, we demonstrated that among patients in POSEIDON Group 1 receiving luteal support with vaginal micronized P plus oral dydrogesterone, the association between serum P and clinical pregnancy rate was significantly modified by BMI. Higher serum P exerted an adverse impact on clinical pregnancy rate in normal-weight patients, whereas this detrimental effect was not observed in overweight individuals. These findings indicate that BMI may act as a critical moderator in the relationship between serum P and pregnancy outcomes, providing novel clinical evidence for individualized luteal support regimens in this specific population.

The mechanistic basis underlying this weight-dependent modification warrants discussion. For normal-weight patients, supraphysiological serum P may disrupt the synchrony between embryo implantation and endometrial development. Excessive serum P in the early luteal phase can accelerate endometrial secretory transformation, potentially advancing the implantation window and desynchronizing embryo-endometrial crosstalk (7). This aligns with the concept of a time-sensitive implantation window and supports the notion that an optimal serum P range, rather than indefinitely high levels, is beneficial for pregnancy success. In contrast, in overweight patients, the observed lack of a significant adverse effect may be attributed to altered P metabolism. Increased adipose tissue mass is associated with changes in P clearance and distribution, which may mitigate the impact of elevated circulating P on the endometrium. Due to the relatively large imbalance in sample size across the three P groups, sensitivity and robustness analyses were further performed. The results of the sensitivity analysis indicated that the independent association between serum P and clinical pregnancy rate remained robust after adjusting for potential confounders, notwithstanding the imbalance in sample sizes in univariate analysis. To further validate the robustness of our findings, BMI-stratified subgroup analyses were performed, and the association trends in all subgroups were consistent with the main analysis, supporting the stability and reliability of the present results.

Our study further defined an optimal range of serum P on the day of ET in POSEIDON Group 1 patients. Both low and high serum P were associated with lower clinical pregnancy rates, suggesting that both insufficient and excessive serum P exposure may impair endometrial receptivity and disrupt embryo-endometrial synchrony. These results are consistent with previous studies reporting that low serum P is associated with unfavorable pregnancy outcomes in HRT cycles (10, 11). However, most previous studies only identified a lower threshold for serum P. The present study further demonstrated that there exists a critical upper threshold for serum P, and that concentrations exceeding 23.32 ng/mL were associated with an adverse effect on clinical pregnancy rate. Notably, some studies (7, 12) reported similar trends but with higher cutoff values (>32.14 ng/mL and >40 ng/mL). This discrepancy may be attributed to the use of a higher daily P dosage, later timing of measurement (2–3 days after ET), or the administration of synthetic progestogens, such as dydrogesterone, which are structurally distinct and cannot be accurately detected by conventional P immunoassays (13). In addition, the discrepancy may also be attributed to differences in study population, sample size, BMI distribution, laboratory protocols, and outcome definitions. Furthermore, our study found that the effect of P was weight-dependent and significant only in normal-weight patients, which may also contribute to the discrepancies between our findings and previous reports.

During the luteal phase of the natural cycle, P is released in a pulsatile manner, leading to marked fluctuations in serum P (14), with variations of up to six-fold within a few hours (15), which compromises the accuracy of serum measurements. In contrast, during artificial cycles, serum P rises rapidly and stabilizes within 24 hours (16), rendering serum measurements more reliable. Studies have demonstrated that only very low serum P levels are required to induce endometrial transformation and support embryo implantation in women of reproductive age (15). Owing to the uterine first-pass effect of vaginal administration, P is preferentially taken up by the endometrium before entering systemic circulation, resulting in significantly higher serum P in the endometrium than in serum. Some scholars have proposed that intrauterine serum P is more critical for successful pregnancy than serum P measurements (5), whereas others have argued that the systemic anti-inflammatory effects of circulating P are equally important in maintaining a favorable immune-endocrine milieu for embryo implantation and placentation (17). According to the latest ESHRE guidelines on ovarian stimulation, any non-oral natural P can be used for luteal phase support, and dydrogesterone is recommended as the only oral alternative with comparable safety and tolerability to other natural P (18). This agent undergoes a unique inversion of its biochemical configuration upon ultraviolet exposure, enabling effective oral administration (19), which provides a rationale for the use of vaginal P combined with dydrogesterone in the present study. However, Metello et al. (20) concluded that supplemental dydrogesterone on the day of ET did not improve the live birth rate. Therefore, the systemic effects of serum P on pregnancy outcomes require further investigation.

An interesting finding of this study was that serum P was negatively associated with BMI. Patients with higher serum P had significantly lower BMI values, which is in line with previous reports (11). This interindividual variation may be attributed to differences in P absorption, distribution, metabolism, and fat storage capacity. In addition, embryo quality and insemination method were important factors affecting pregnancy outcomes in this study. The higher clinical pregnancy rate in the ICSI group may be mainly related to male factor infertility, in which ICSI effectively overcomes fertilization disorders (21). Nevertheless, the relatively small number of ICSI patients in this study may introduce potential selection bias, and this result should be interpreted cautiously.

Several limitations of this retrospective single-center study should be acknowledged. First, potential selection bias and the single-center design may limit the generalizability of our findings. Second, only a single serum P measurement on the day of ET was analyzed, without dynamic monitoring. Third, the relatively small sample size—especially after BMI stratification—led to insufficient statistical power for secondary outcomes (e.g., live birth rate). Fourth, PGT application could not be adjusted due to incomplete data. Additionally, standard P assays cannot detect dydrogesterone, so the measured levels may not fully reflect total progestational exposure. Finally, unmeasured or residual confounding factors cannot be entirely excluded. Thus, these results are exploratory in nature, and further large-scale prospective studies are required to verify our conclusions.

In conclusion, among POSEIDON Group 1 patients undergoing HRT cycles with vaginal micronized P combined with oral dydrogesterone, an optimal serum P range on the day of ET is associated with the highest clinical pregnancy rate. High serum P is independently associated with decreased clinical pregnancy rates in normal-weight patients but not in overweight patients. BMI status significantly modulates the effect of P on pregnancy outcomes. These findings support the implementation of individualized luteal support based on serum P and BMI stratification to improve reproductive outcomes in this specific population.

## Data Availability

The original contributions presented in the study are included in the article/**Supplementary Material**. Further inquiries can be directed to the corresponding author.
