# Supplementary material for: Association between serum progesterone levels on the day of embryo transfer and clinical pregnancy outcomes in POSEIDON Group 1 patients
Source: Front Endocrinol (Lausanne). 2026 May 28;17:1766243. doi: 10.3389/fendo.2026.1766243 (PMC13253454; doi:10.3389/fendo.2026.1766243)
Supplement: Supplementary file 1 [file DataSheet1.pdf]

STROBE Statement—Checklist of items that should be included in reports of ***cohort studies***

	Item No	Recommendation
**Title and abstract**	1	(*a*) **Title**:Association between Serum Progesterone Levels on the Day of Embryo Transfer and Clinical Pregnancy Outcomes in POSEIDON Group 1 Patients
		(*b*) **ABSTRACT**<br><br>Objective:To investigate the relationship between serum progesterone level on embryo transfer day and pregnancy outcomes.<br>Methods: A retrospective cohort study was performed at the Reproductive Center of Yantai Yuhuangding Hospital from January 2016 to December 2023, enrolling 402 patients undergoing day-3 or day-5 single or double embryo transfer. Serum progesterone and estradiol levels were detected on the day of embryo transfer, with clinical pregnancy rate as the primary outcome. Patients were categorized into three groups according to the 10th and 90th percentiles of serum progesterone levels. Categorical variables were compared via Pearson chi-square test or Fisher's exact test, continuous variables by Student's t-test, and multiple quantitative data by one-way analysis of variance. Multivariate logistic regression was applied to adjust for confounders. Moreover, sensitivity analysis and BMI stratification-based robustness test were conducted.<br>Results:The 10th and 90th percentiles of serum progesterone on embryo transfer day were 9.45 ng/mL and 23.32 ng/mL,respectively.The clinical pregnancy rate peaked in the 9.45-23.32 ng/mL group and decreased significantly when progesterone exceeded 23.32 ng/mL. Multivariate logistic regression showed that high progesterone (⩾23.32 ng/mL) was independently associated with a lower clinical pregnancy rate after adjustment for confounders(adjusted OR = 1.454, 95% CI: 1.032–2.048, P = 0.032). Sensitivity analysis confirmed the robustness of this association. BMI stratification revealed that elevated progesterone was associated with a lower clinical pregnancy rate in normal-weight patients but not in overweight/obese patients, with a significant interaction between progesterone and BMI.<br>Conclusions:Serum progesterone level on embryo transfer day was significantly associated with clinical pregnancy rate in POSEIDON Group 1 patients, and BMI significantly modified this association. High progesterone levels were associated with lower clinical pregnancy rate in normal-weight patients but not in overweight patients. These findings support individualized luteal support based on progesterone level and BMI stratification.<br>Keywords: serum progesterone; clinical pregnancy rate; embryo transfer; vaginal progesterone; dydrogesterone
**Introduction**		
Background/rationale	2	In recent years,with the improvement of cryopreservation embryo technology and the increase of patients undergoing Preimplantation Genetic Testing(PGT) and fertility preservation,the number of frozen embryo transfers(FET) is increasing around the world.At the same time,the risk of late-onset OHSS in fresh cycle and the adverse effects of high estrogen levels on endometrial receptivity were reduced[1].Progesterone is necessary for endometrial transformation,embryo implantation and maintenance of pregnancy.Compared to the natural and the stimulated cycles,the artificial endometrium preparation cycles with hormonal replacement therapy (HRT) is more popular.Because it can control the number of days of exposure to exogenous progesterone and accurately determine the transplantation time,to synchronize the embryo with endometrial development[2].Therefore,there is no endogenous progesterone release in the artificial cycle,so the supplement of exogenous progesterone is the key to the success of embryo transfer.HRT mimics the natural cycles,supplementing estrogen from the 2nd to 3rd day of menstruation,until the endometrium reaches 8mm,and progesterone conversion is performed.<br>It has been found that in the natural cycles,the increase of progesterone before ovulation leads to premature luteinization and impaired endometrial receptivity,and the

		implantation rate is reduced[3].However,insufficient progesterone supplementation before embryo transfer(ET) also adversely affects the implantation rate[4].Currently,progesterone can be administered by different routes,including vaginal,intramuscular,subcutaneous,rectal or oral routes,with different pharmacokinetics.Some scholars believe that intrauterine progesterone levels are more critical to pregnancy success than serum P[5].However,some studies have shown that the systemic anti-inflammatory effects of serum P are equally important[6],and progesterone in the endometrium is derived from arterial blood [7].At present,there is no consensus on the optimal route,dose and duration of progesterone administration of FET,which directly affect the serum P levels on the day of ET.Previous studies have suggested that the higher the levels of serum P on the day of ET,the better the pregnancy outcome[8].However,some studies have found that there is an optimal threshold of serum P on the day of transplantation,and too low or too high progesterone also has adverse effects on pregnancy outcomes[9].
Objectives	3	We aimed to evaluate the association between serum progesterone levels on the day of embryo transfer and pregnancy outcomes. The present study specifically focused on POSEIDON Group 1 patients undergoing hormone replacement therapy (HRT) cycles with luteal support provided by vaginal micronized progesterone combined with oral dydrogesterone, in order to provide evidence for individualized luteal support strategies in this population.
**Methods**		
Study design	4	This study is a retrospective cohort study.
Setting	5	A retrospective cohort study was performed at the Reproductive Medicine Center of Yantai Yuhuangding Hospital. Participants were patients undergoing frozen embryo transfer (FET) following hormone replacement therapy (HRT) cycles from January 2016 to December 2023.
Participants	6	A total of 402 POSEIDON Group 1 patients undergoing hormone replacement therapy (HRT) frozen embryo transfer cycles with luteal support by vaginal micronized progesterone plus oral dydrogesterone were included in this retrospective cohort study
Variables	7	A total of 7 key variables were analyzed, including serum progesterone and estradiol levels on embryo transfer day, body mass index (BMI), clinical pregnancy rate, age, anti-Müllerian hormone (AMH), and number of high-quality embryos transferred.
Data sources/ measurement	8*	Data were obtained from the electronic medical record system of the Reproductive Center of Yantai Yuhuangding Hospital. Serum progesterone and estradiol levels were measured on the day of embryo transfer using a chemiluminescent immunoassay. Other variables, including age, BMI, AMH, endometrial thickness, and embryo characteristics, were extracted from the medical records.
Bias	9	Selection bias was minimized by consecutively enrolling all eligible patients during the study period. Information bias was reduced by using standardized medical record extraction and laboratory measurement protocols. Residual confounding was addressed by multivariate logistic regression adjustment.
Study size	10	A total of 402 POSEIDON Group 1 patients were included. The sample size was determined based on the available data during the study period, and a post-hoc power analysis confirmed sufficient statistical power to detect the observed interaction effect between progesterone and BMI.
Quantitative variables	11	Quantitative variables were described as mean ± standard deviation (if normally distributed) or median (interquartile range) (if non-normally distributed). These included age, BMI, AMH, serum progesterone and estradiol levels on embryo transfer day, endometrial thickness, number of oocytes retrieved, and number of high-quality embryos transferred. Between-group differences were analyzed using the Student's t-test or Mann-Whitney U test as appropriate.

Statistical methods	12	Serum progesterone (P) levels on the day of ET were categorized according to the 5th, 10th, 25th, 50th, 75th, 90th, and 95th percentiles. Patients were further divided into three groups based on serum P levels, with the 10th and 90th percentiles used as cutoff values.Categorical variables were compared among groups using the Pearson chi-square test or Fisher's exact test. The Student's t-test was used for comparisons of continuous variables between two groups, and one-way analysis of variance (ANOVA) was applied for comparisons among multiple groups.Clinical pregnancy rate, biochemical pregnancy rate, live birth rate, and spontaneous abortion rate were compared among the three groups.We used the 10th (P10) and 90th (P90) percentiles of serum progesterone distribution as cutoff values to stratify patients into three groups.As univariate analysis showed no significant difference in progesterone levels between pregnant and non-pregnant patients, a valid and clinically meaningful cutoff could not be determined by ROC curve analysis.Furthermore, progesterone levels on the day of ET mainly reflect interindividual variability in medication absorption, rather than acting as a diagnostic index with definitive sensitivity and specificity.For these reasons, we adopted percentile-based stratification for subsequent analyses.<br>Multivariate logistic regression analysis was conducted to explore the independent effects of serum progesterone (P) levels on clinical pregnancy rate and identify independent factors associated with clinical pregnancy. The regression model was adjusted for comprehensive potential confounders, including age, BMI, AMH level, type and duration of infertility, number of oocytes retrieved in the fresh cycle, insemination method in the fresh cycle, endometrial thickness, number embryos transferred(single versus double), embryo stage(cleavage versus blastocyst),number of high-quality embryos transferred, and serum estradiol (E2) level. In the binary logistic regression model, continuous variables were directly entered into the model, while categorical variables were converted into dummy variables with a predefined reference group before analysis..Sensitivity analysis was conducted by combining the low and high progesterone groups and comparing them with the middle group.In addition, robustness checks and interaction analyses were performed with body mass index (BMI) as a stratification or interaction variable to validate the stability of the main results.
**Results**		
Participants	13*	A total of 402 POSEIDON Group 1 patients undergoing hormone replacement therapy (HRT) frozen embryo transfer cycles with luteal support by vaginal micronized progesterone plus oral dydrogesterone were included in this retrospective cohort study
Descriptive data	14*	Baseline clinical characteristics of the study population are summarized in Table III. Continuous variables are presented as mean ± standard deviation (SD), and categorical variables are presented as number (percentage).Among the 402 patients included, the mean age was 30.70±2.42 years, mean BMI was 24.34±3.91 kg/m², and mean AMH level was 5.41±4.58 ng/mL. Primary infertility accounted for 64.18% of the cohort, and the mean duration of infertility was 3.65±2.05 years.
Outcome data	15*	The primary outcome was clinical pregnancy rate, defined as the presence of a gestational sac on ultrasound at 6–7 weeks of gestation. Secondary outcomes included live birth rate, biochemical pregnancy rate, and spontaneous abortion rate. All outcomes were extracted from the electronic medical records.
Main results	16	Clinical pregnancy rate peaked in the intermediate progesterone group (9.45–23.32 ng/mL) and was significantly lower in the high-progesterone group (≥23.32 ng/mL) after adjustment for confounders (adjusted OR = 0.397, 95% CI: 0.188–0.837, P = 0.015). BMI stratification revealed a significant interaction: high progesterone was associated with reduced clinical pregnancy in normal-weight patients but not in overweight/obese patients (P for interaction = 0.045).

Other analyses	17	Sensitivity analysis combining low- and high-progesterone groups confirmed the robustness of the association between progesterone levels and clinical pregnancy rate (adjusted OR = 1.745, 95% CI: 1.026–3.066, P = 0.040). Subgroup analyses by insemination method and embryo quality were also performed, with consistent findings observed across subgroups.
**Discussion**		
Key results	18	In the present study, we demonstrated that among patients assigned to Poseidon Group 1 undergoing luteal support with vaginal micronized progesterone plus oral dydrogesterone, the association between serum progesterone levels and clinical pregnancy rate was significantly modified by body weight status. Higher progesterone concentrations exerted an adverse impact on clinical pregnancy rate in normal-weight patients, whereas this detrimental effect was not observed in overweight individuals.These findings highlight that body weight may act as a critical moderator in the relationship between progesterone levels and pregnancy outcomes, providing novel clinical evidence for individualized luteal support regimens in this specific population.
Limitations	19	Several limitations of this retrospective single-center study should be acknowledged. First, potential selection bias and single-center design may limit the generalizability of our findings. Second, only a single progesterone measurement on the day of embryo transfer was analyzed, without dynamic monitoring. Third, the relatively small sample size—especially after BMI stratification—led to insufficient statistical power for secondary outcomes (e.g., live birth rate). Fourth, PGT use could not be adjusted for due to incomplete data. Additionally, standard progesterone assays do not detect dydrogesterone, so measured levels may not fully reflect total progestational exposure. Finally, unmeasured or residual confounding cannot be entirely excluded. Thus, these results are exploratory, and further large-scale prospective studies are needed to verify our conclusions.
Interpretation	20	Our findings indicate an optimal range of serum progesterone (9.45–23.32 ng/mL) for clinical pregnancy in POSEIDON Group 1 patients, with levels >23.32 ng/mL associated with adverse outcomes. Importantly, BMI modifies this association: high progesterone impairs pregnancy in normal-weight patients but not in overweight/obese individuals, suggesting the need for personalized luteal support.
Generalisability	21	While our single-center, retrospective design limits the direct generalizability of these findings to broader populations, the observed association between elevated progesterone levels and compromised pregnancy outcomes in normal-weight patients warrants cautious consideration in clinical practice. Further multi-center prospective studies are needed to confirm these observations and clarify their applicability across different patient subgroups and luteal support protocols
**Other information**		
Funding	22	No funding was received for this study.

*Give information separately for exposed and unexposed groups.

Note: An Explanation and Elaboration article discusses each checklist item and gives methodological background and published examples of transparent reporting. The STROBE checklist is best used in conjunction with this article (freely available on the Web sites of PLoS Medicine at http://www.plosmedicine.org/, Annals of Internal Medicine at http://www.annals.org/, and Epidemiology at http://www.epidem.com/). Information on the STROBE Initiative is available at http://www.strobe-statement.org.
